# Spatial Updating Depends on Gravity

**DOI:** 10.3389/fncir.2020.00020

**Published:** 2020-06-05

**Authors:** Alexander Christoph Stahn, Martin Riemer, Thomas Wolbers, Anika Werner, Katharina Brauns, Stephane Besnard, Pierre Denise, Simone Kühn, Hanns-Christian Gunga

**Affiliations:** ^1^Department of Psychiatry, Perelman School of Medicine at the University of Pennsylvania, Philadelphia, PA, United States; ^2^Charité—Universitätsmedizin Berlin, Corporate Member of Freie Universität Berlin, Humboldt-Universität zu Berlin, and Berlin Institute of Health, Institute of Physiology, Berlin, Germany; ^3^Aging and Cognition Research Group, German Center for Neurodegenerative Diseases (DZNE), Magdeburg, Germany; ^4^Normandie Université, UNICAEN, INSERM, COMETE, Caen, France; ^5^Department of Psychiatry and Psychotherapy, University Medical Center Hamburg-Eppendorf, Hamburg, Germany; ^6^Lise Meitner Group for Environmental Neuroscience, Max Planck Institute for Human Development, Berlin, Germany

**Keywords:** spatial navigation, spatial updating, precuneus, weightlessness, vestibular system, parabolic flight, spaceflight

## Abstract

As we move through an environment the positions of surrounding objects relative to our body constantly change. Maintaining orientation requires spatial updating, the continuous monitoring of self-motion cues to update external locations. This ability critically depends on the integration of visual, proprioceptive, kinesthetic, and vestibular information. During weightlessness gravity no longer acts as an essential reference, creating a discrepancy between vestibular, visual and sensorimotor signals. Here, we explore the effects of repeated bouts of microgravity and hypergravity on spatial updating performance during parabolic flight. Ten healthy participants (four women, six men) took part in a parabolic flight campaign that comprised a total of 31 parabolas. Each parabola created about 20–25 s of 0 g, preceded and followed by about 20 s of hypergravity (1.8 g). Participants performed a visual-spatial updating task in seated position during 15 parabolas. The task included two updating conditions simulating virtual forward movements of different lengths (short and long), and a static condition with no movement that served as a control condition. Two trials were performed during each phase of the parabola, i.e., at 1 g before the start of the parabola, at 1.8 g during the acceleration phase of the parabola, and during 0 g. Our data demonstrate that 0 g and 1.8 g impaired pointing performance for long updating trials as indicated by increased variability of pointing errors compared to 1 g. In contrast, we found no support for any changes for short updating and static conditions, suggesting that a certain degree of task complexity is required to affect pointing errors. These findings are important for operational requirements during spaceflight because spatial updating is pivotal for navigation when vision is poor or unreliable and objects go out of sight, for example during extravehicular activities in space or the exploration of unfamiliar environments. Future studies should compare the effects on spatial updating during seated and free-floating conditions, and determine at which g-threshold decrements in spatial updating performance emerge.

## Introduction

Gravity is critical for various physiological functions and goal-directed behavior. The lack of gravity, i.e., weightlessness, leads to cardiovascular deconditioning, negative energy balance, bone and muscle loss, and sensorimotor impairments. The time course of these processes varies considerably between immediate effects upon entry to weightlessness and long-term effects occurring after several weeks to months of space travel (Nicogossian et al., 2016).

One system that is immediately affected by gravity is the vestibular system. The vestibular system senses linear and angular acceleration through signals from the otoliths and semicircular canals, and it drives various reflexes such as keeping gaze and posture when linear accelerations are changing. However, the vestibular system goes beyond maintaining gaze and balance. Interactions between the otoliths and semicircular canals critically contribute to spatial perception including self-motion, orientation, and navigation (Cullen and Taube, 2017). During weightlessness gravity no longer acts as a fundamental reference, and the discrepancy between vestibular (including conflicts between otolith and semicircular canal information), visual, and sensorimotor signals can affect spatial abilities (Clément et al., 1989; McIntyre et al., 2001). So far, microgravity research has concentrated on posture, gaze, functional mobility, and spatial orientation, reporting misperceptions of visual orientation, depth and distance, and difficulties in shape recognition (Reschke and Clément, 2018). Whether the lack of gravity also impairs spatial navigation performance and strategies is not well understood.

Spatial navigation is an essential cognitive process that allows us to perceive our position in the environment and use this information to efficiently move in physical space. A fundamental component of spatial navigation requires to continuously form and update transient sensorimotor representations about self-to-object relations during locomotion. This ability has been termed spatial updating and is closely linked to working memory (Wolbers et al., 2008; Theeuwes et al., 2009; Anderson et al., 2010). It requires special effort when objects are no longer visible (Boon et al., 2018) and is a prerequisite for route learning and wayfinding in large-scale space. Spatial updating is also vital for navigation in small-scale spaces when vision is poor or unreliable and objects go out of sight, for example, during extravehicular activities during spaceflight operations and exploration of unfamiliar planets. Given that the perception of self-motion critically depends on integrating visual information with gravitational cues processed by the vestibular system (Pfeiffer et al., 2014), spatial updating performance could be expected to be impaired when gravity levels change. This prediction is also in line with emerging evidence highlighting the cortical projections of the vestibular system. This includes several brain regions important for spatial navigation, including the hippocampal and parahippocampal formation, cingulate gyrus and retrosplenial cortex, parietal and medial temporal cortices, and the parietoinsular vestibular cortex and temporoparietal junction (Hitier et al., 2014).

**Figure 1:**
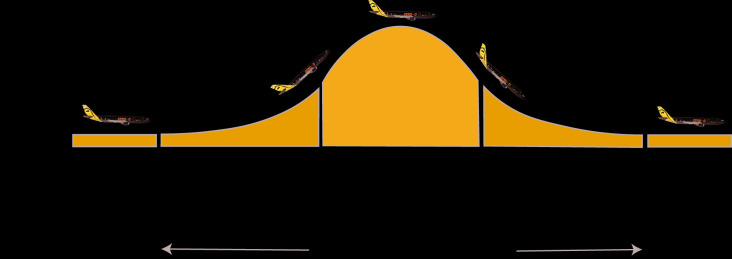
The characteristic profile of a parabolic flight maneuver. At standard cruising altitude (about 12,000 ft) the aircraft is pulled up at a 47° angle, inducing a gravito-inertial acceleration (GIA) of 1.5–1.8 g, after which the engine’s thrust is limited to compensate air-drag, entering a phase of free-fall comparable to 0 g, and hence, weightlessness. This phase is completed by another phase of hypergravity before returning to 1 g again. After an initial test parabola, the maneuver is repeated a total of 30 times with 3–5 min breaks between parabolas and a longer (about 8 min) break after the 16th parabola.

Here, we tested the effects of different gravity levels on spatial updating performance using a parabolic flight maneuver. A parabolic flight maneuver starts with a hypergravity phase (1.8 g) of about 20 s, after which the aircraft enters a free-fall state for about 20–25 s that is comparable to 0 g because of the lack of ground reaction forces. The period of weightlessness is followed by another hypergravity phase before reaching 1 g again (Figure 1). This maneuver was performed a total of 31 times, with the first parabola being a test parabola, where no data were collected. Subsequently, six blocks of parabolas were performed, separated by 5-min to 8-min breaks. Each parabola within blocks was separated by 2-min to 3-min breaks, yielding a total of about 12 min of weightlessness. Spatial updating performance was assessed at normal Earth gravity (1 g), hypergravity (1.8 g) and zero-g (0 g). The paradigm was specifically designed to meet the requirements of the parabolic flight maneuver and to allow differentiating between changes in spatial working memory performance (static condition) and spatial updating performance (updating condition). During the updating condition participants had to first encode two egocentric object locations, then update these positions during a virtual translational forward movement when the objects were no longer visible and finally point back to the location of one of the original objects after completion of the forward movement. In the static condition, the task was identical except that the participants did not experience the virtual translation, eliciting working memory processes without the need to update egocentric object locations. We hypothesized that compared to 1 g, both 0 g and 1.8 g would selectively impair spatial updating.

## Materials and Methods

### Participants

A total of 10 healthy adults (four women, six men, aged 33–50 years, mean ± SD: 39 ± 4 years) with no previous parabolic flight experience participated in the experiment. All participants had normal or corrected-to-normal vision and underwent a medical aptitude screening to participate in a parabolic flight. Subjects gave informed written consent to participate in the study. The study was approved by the local Ethics Committee of Charité—Universitätsmedizin Berlin, by the European Space Agency (ESA) medical board, and by the French Ethics Committee—Comité de Protection des Personnes (CPP Nord-Ouest III) and authorized by the French Competent Authority (ANSM). All procedures were conducted following the principles of the Declaration of Helsinki.

### Procedure

#### Parabolic Flight

The experiment was conducted as part of the European Space Agency (ESA) 66th parabolic flight campaign in May 2017. The flights were performed by Novespace^1^ using a modified Airbus A 310 aircraft, i.e., the Airbus A 310 Zero-G, based at Bordeaux-Merignac International Airport in France. The campaign consisted of three consecutive flight days. Three participants were flown on the first and second flight day, and four on the third flight day. Each flight consisted of 31 parabolic maneuvers. During each maneuver the aircraft started from a regular horizontal flight at typical flight altitude and pulls up to an angle of 47°, producing a gravito-inertial acceleration (GIA), defined as the sum of gravity’s linear acceleration and inertial forces, of 1.5–1.8 g. After about 20 s the engine’s thrust was reduced just to compensate air-drag, and the aircraft enters a freefall trajectory for 20–25 s. During this period the aircraft and all materials and passengers in the plane fell at 9.81 m/s^2^, achieving a net 0 g-level. We acknowledge that the terms weightlessness is technically not correct to describe this phase. Gravity is still 1 g throughout the entire flight. This is similar to the condition on the International Space Station (ISS), where gravity is still >90% of Earth’s gravity, but astronauts experience a constant free fall due to the station’s orbit around the Earth. Despite this discrepancy, we follow the typical convention in Space Life Sciences and consider the condition of a net level of 0 g during the free fall as weightlessness. This phase was ended by gradually pulling the aircraft out of the freefall, inducing another hypergravity phase of 1.5–1.8 g before returning to a horizontal flight position again. The aircraft pitch rotation (about 3°/s) is barely detectable by the vestibular system (Karmali and Shelhamer, 2008). Each flight including take-off and landing took about 3.5 h.

#### Data Acquisition

**Figure 2:**
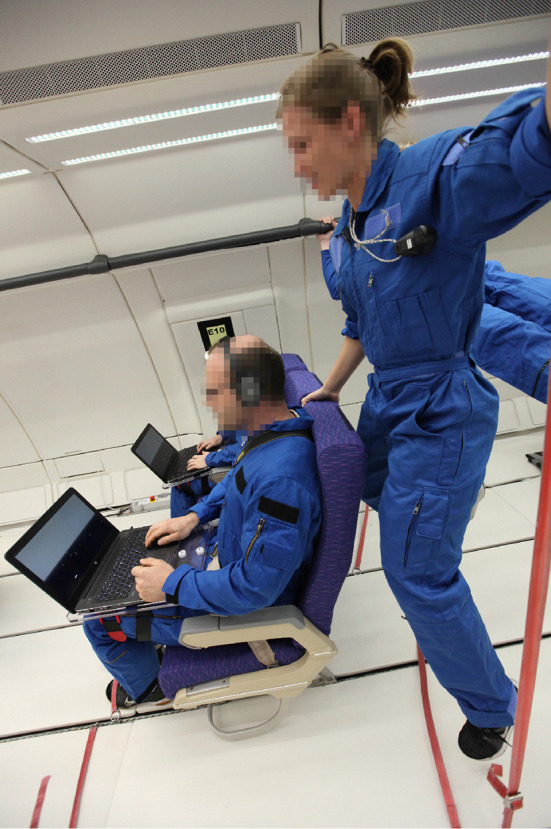
Experimental setup in the Airbus A 310 Zero-G. Participants were buckled up in standard aircraft chairs with feet fixed to their ground floor with foot straps. The laptops were mounted to a plexiglass plate that was strapped to the participants’ upper legs that allowed them to maintain the same position throughout testing. Testing was performed during 15 parabolas, providing a total of 90 trials (30 trials during 1 g, 1.8 g, and 0 g, respectively). Photo credit: Novespace/ESA.

All participants completed two training sessions on the ground. Both training sessions were performed onsite at Novespace. The first session was performed at the facilities of Novespace, and the second training session was performed in the aircraft using the identical setup that was used during the flight. About 75–90 min before take-off all but one participant received scopolamine subcutaneously to minimize motion-sickness (about 0.125 mg for women and 0.175 mg for men). Inflight testing was performed either between the 2nd and 16th parabola or between the 17th and 31st parabola. Participants were randomly allocated to the order in a balanced fashion. During the remaining parabolas, participants were allowed to free float in a designated area at the end of the aircraft. Before the first, after the 16th and 31st parabola subjects were asked to indicate their current level of motion sickness on a 5-point Likert scale with the two anchors “not at all” (1) and “very sick” (5). During testing, subjects were seated and buckled up in standard aircraft chairs with their feet fixed to the ground floor with foot straps (see Figure 2). The laptops were mounted to a plexiglass plate that was strapped to the participants’ upper legs that allowed them to maintain the same position throughout testing. During each 1 g phase, hypergravity phase, 0 g phase, and following the completion of the parabolic maneuver participants performed a block of two trials, respectively. Accordingly, a total of 30 trials (15 parabolas x 2 trials) were performed per gravity level. The 30 trials in each phase comprised 6 static trials, 12 spatial updating trials involving a short forward motion, and 12 spatial updating trials involving a long forward motion (see details below). To ensure exact timing during all phases, each block was started by an experimenter who also verbally instructed the participants when each block was started.

### Experimental Stimuli and Paradigm

**Figure 3:**
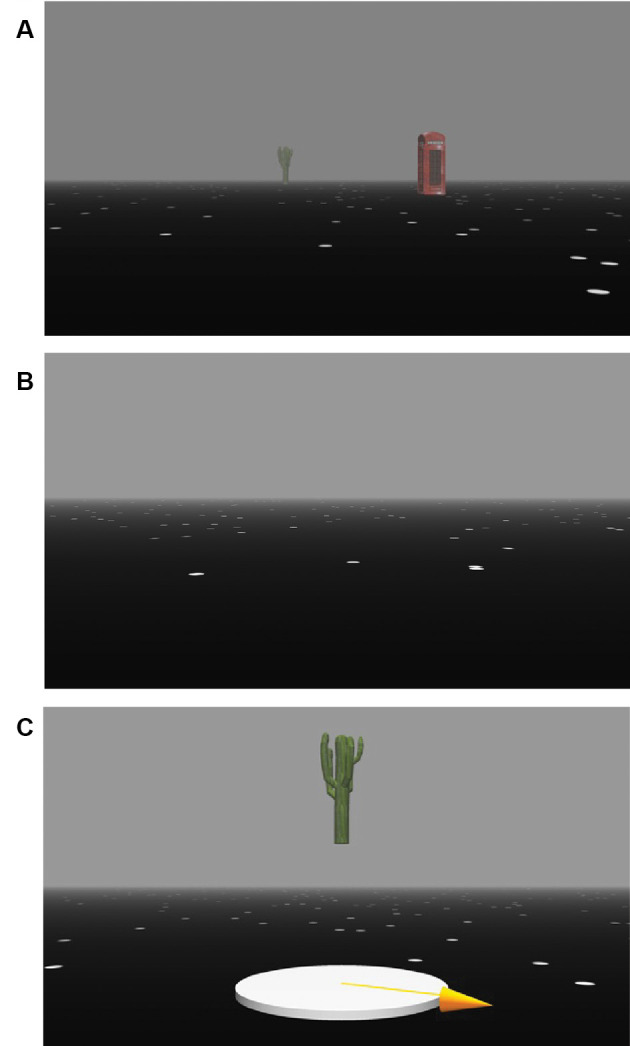
Experimental paradigm. Trials comprise an encoding phase **(A)**, a delay phase **(B)**, and a retrieval phase **(C)**. Each trial started with a static presentation of the virtual environment and two objects located at two different positions during which participants had to memorize the location and identity of the objects. Next, all objects gradually sank into the ground until they completely disappeared. In the delay phase, participants either experienced a forward movement of 25 m or 45 m (updating trials) or remained at their position (static trials). In the subsequent retrieval phase, one of the two objects was presented in the center of the screen, and participants had to turn a 3D-arrow towards the object’s original position in the encoding phase.

Spatial updating performance was assessed with a paradigm that specifically targets the precuneus (Wolbers et al., 2008). The paradigm was modified to meet the demands of parabolic flight characteristics, programmed in Vizard 5 (WorldViz, Santa Barbara, CA, USA), and presented on a 15-inch laptop (ZBook 15 G5 Mobile Workstation, Hew). Participants saw a virtual three-dimensional (3D) environment from a first-person perspective (eye height: 180 cm). The ground surface consisted of white limited life-time dots randomly fading and appearing (maximal duration: 5 s). Each trial comprised an encoding, a delay, and a retrieval phase. In the encoding phase, participants were presented with two different objects positioned at distances between 15 and 55 m in front of them, one object to the left and one to the right of the participant. The target object locations were the same in all g-levels, but they were presented in randomized order (different randomization for all g-levels). The objects were of similar size typically encountered in everyday life such as a lamppost, a road sign, a phone booth or a statue (Figure 3). Participants were instructed to memorize the object locations as precisely as possible. After 1 s of the presentation, the objects sank into the ground. After another second, participants were passively moved forward at a uniform velocity of 8.3 m/s and 15 m/s for short and long updating trials, respectively, or they remained at their original position (static trials). The updating trials consisted of a short or long updating period (forward motion of either 25 or 45 m). For all trials, the duration was kept constant at 3 s to eliminate potential influences of time-keeping mechanisms (Riemer et al., 2014). The delay phase in static trials was also set to 3 s. In the final retrieval phase, an image of one of the two objects shown in the encoding phase was presented at the center of the screen, and participants were asked to turn a 3D-arrow to indicate the direction of the target object’s location. The arrow was controlled with the left and right arrow keys of the keyboard and responses were logged with the space bar. The initial orientation of the arrow was always pointing forward for all trials to reduce error variance. No feedback was given, and trials were separated by a black screen with an intertrial interval of 1 s.

### Behavioral and Statistical Analysis

We recorded reaction time (RT) and response pointing angle for each trial. Pointing error was defined as our primary outcome and calculated as the difference between the correct angle and response pointing angle. To assess outliers, we first computed the number of signed pointing errors exceeding 1.5 times the interquartile range (IQR). The number of these potential outliers were then used to perform Rosner’s generalized extreme studentized deviate test to confirm outliers that were removed from further analysis. Pointing performance is characterized by perceptual error and noise (Wolbers et al., 2008). The former is associated with the encoding of the object locations in a rather non-immersive virtual environment projected on a 2D screen. However, given that the positions of the target objects were identical across all conditions, any increase in pointing error would reflect increases in noise. To quantify this noise, we computed variable pointing error as the standard deviation of the signed pointing errors across trials for each participant, phase, and condition using circular statistics (Fisher, 1993). High performance is therefore demonstrated by small differences in pointing errors between trials. In contrast, large differences in pointing errors between trials suggest a high uncertainty of pointing performance (Wolbers et al., 2008). Hence, increases in variable pointing errors during 0 g and 1.8 g were expected to reflect impairments in working memory processes associated with the updating of self-to-object relations. We also determined accuracy, i.e., the mean direction of pointing errors calculated as the circular mean of signed pointing errors. Differences between task conditions and g phases were assessed using mixed linear models with g-level and task condition as fixed factors, and subjects as a random factor with random intercepts and random slopes for condition (random slopes were not fitted if the model did not converge). Pre-planned contrasts were used to compare the levels within each factor using Bonferroni-corrected family-wise comparisons (considering each main factor as one family). We first assessed the effect of task condition on pointing performance by comparing static to short and long updating trials and short to long updating trials at each g-level (correction for a total of three comparisons). We then compared 1 g to 0 g, 1 g to 1.8 g, and 0 g to 1.8 g for each task condition (correction for a total of three comparisons). We also assessed the effects of g-levels on RT. No comparisons of RT were performed between task conditions because they were confounded by movement times of the pointing indicator due to the nature of the paradigm. Recall that the pointing indicator displayed in the retrieval phase was always parallel to the direction of the forward translation (see also “Experimental Stimuli and Paradigm” section). Consequently, RTs were necessarily affected by task conditions because the arrow had to be moved a shorter angular distance for static and short updating trials compared to long updating trials. For these reasons, we performed mixed models for RT separately for each task condition using g-level as a fixed factor and subjects as a random factor. Pre-planned contrasts were performed to assess the differences between 1 g and 0 g, 1 g and 1.8 g, and 0 g and 1.8 using Bonferroni-corrected family-wise comparisons (correction for a total of three comparisons). Effect sizes for contrasts were expressed as Cohen’s *d* with Bonferroni-adjusted 95% confidence intervals using bootstrapping (Kirby and Gerlanc, 2013). To assess systematic variations in pointing performance throughout the flight we identified the association between absolute pointing error and trial number for each g-level and condition using repeated measures correlations (*r*_rm_; Roy, 2006). All statistical analyses and graphical illustrations were carried out using the software package R (R Core Team, 2016).

## Results

None of the participants experienced discomfort and all demonstrated excellent compliance during the task. The level of motion sickness did not change significantly (mean change (95% CI): before 1st vs. after 16th parabola: 0.4 (−1.09, 0.29), *P* = 0.22; before 1st vs. after 31th parabola: 0.45 (−1.32, 0.42), *P* = 0.27). On average only two trials (out of 90) were missed (range: 0 to 6 trials). One hundred and two pointing responses (11.6%) were identified as outliers using boxplot statistics (1.5 × IQR). A Rosner test with a maximum of 102 potential outliers revealed 12 extreme outliers (1.4%) that were excluded from further analysis. Neither condition nor phase was missing more than two responses with a maximum of seven missing responses in total for any participant. The final data set included 864 pointing responses (mean: 86; range: 83 to 90 per participant). All repeated measures correlations between trial number and mean absolute pointing error were minimal and non-significant for all g-levels and task conditions (*r*_rm_ = −0.12, *P* = 0.24 to *r*_rm_ = 0.15, *P* = 0.32), confirming that there were no learning or habituation effects in pointing performance throughout the experiment.

**Figure 4:**
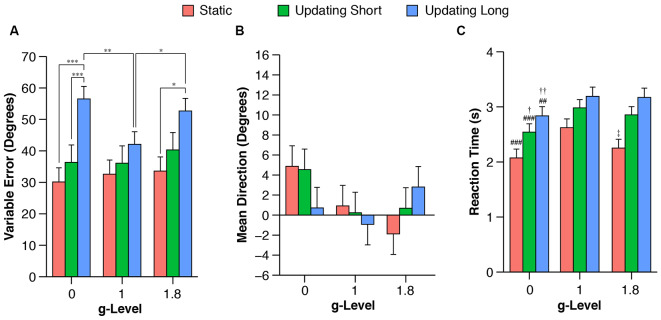
Mean variable **(A)** and signed **(B)** pointing errors, and reaction time (RT; **C**) during 0 g, 1 g, and 1.8 g and different trial conditions (static, updating short, updating long). Variable pointing error was computed as the standard deviation of the signed pointing errors for each participant and each g-level and task condition using circular statistics. Data are estimated means and standard errors. Note that no contrasts were performed between task conditions for RTs because RTs were a logical consequence of task condition, i.e., the 3D arrow had to be moved a shorter angular distance for static and short updating trials compared to long updating trials (for details see “Behavioral and Statistical Analysis” in “Materials and Methods” section). *P < 0.05. **P < 0.01. ***P < 0.001. ^##^*P* < 0.01 for 0 g vs. 1 g. ^###^*P* < 0.01 for 0 g vs. 1 g. ^†^*P* < 0.05 for 0 g vs. 1.8 g. ^††^*P* < 0.01 for 0 g vs. 1.8 g. ^‡^*P* < 0.05 for 1 g vs. 1.8 g.

Pointing performance was affected by task condition (*F*_(2,72)_ = 10.6, *P* < 0.001). A trend toward significance was observed for the prediction of g-level on pointing performance (*F*_(2,72)_ = 2.5, *P* = 0.089) and the interaction between g-level and task condition (*F*_(2,72)_ = 2.3, *P* = 0.07). Across all g-levels variable pointing error increased from static to short, and from short to long updating trials (Figure 4). Planned contrasts revealed significant differences of pointing performance at 0 g between static and long updating trials [*t*_(72)_ = 4.06, *P* < 0.001; *d* = 1.53 (0.81, 2.88)] and between short and long updating trials [*t*_(72)_ = 3.91, *P* < 0.001; *d* = 1.12 (0.07, 2.33)]. Similarly, we found a significant difference between static and long updating trials [*t*_(72)_ = 2.94, *P* = 0.013, *d* = 0.73 (−0.33, 1.57)] and a nearly significant difference between short and long updating trials [*t*_(72)_ = 2.4, *P* = 0.056; *d* = 0.8 (0.03, 1.58)] in the 1.8 g condition. We also observed a stepwise increase in pointing error from static to short updating to long updating trials. These differences did not reach the level of significance (Table 1) because of the somewhat smaller differences in variable pointing error. For instance, we observed a difference between long and static conditions of 26.4° and 19.1° for 0 g and 1.8 g compared to 9.5° for 1 g. We performed a power analysis using the R package pwr and found that a sample size of *N* = 36 would have been needed to detect a significant difference of 9.5° between static and long updating trials during 1 g. Together, these data suggest that long updating trials were particularly more challenging compared to the static or short updating trials, and these effects were most pronounced during 0 g and 1.8 g. Variable pointing error for long updating trials was significantly higher during 0 g compared to 1 g [*t*_(72)_ = 3.37, *P* < 0.01; *d* = 0.94 (0.07, 2.08)], and during 1.8 g compared to 1 g [*t*_(72)_ = 2.48, *P* = 0.047; *d* = 0.66 (−0.21, 1.87)]. Neither static nor short updating trials revealed any significant differences of pointing error between any g-levels (Table 2), indicating that gravity affected spatial updating pointing performance in complex (i.e., long) trials and this effect was strongest during 0 g. Mean signed pointing error was larger in both 0 g and 1 g, but neither the main effects (g-level: *F*_(2,72)_ = 2.8, *P* = 0.07; task condition: *F*_(2,72)_ = 0.2, *P* = 0.82) nor their interaction reached statistical significance (*F*_(4,72)_ = 1.6, *P* = 0.18).

**Table 1:** Contrasts examining the effects between static (Static), short updating (Short), and long updating (Long) task conditions on variable pointing error (Var PE) and mean direction of signed pointing error (Mean PE) during 0 g, 1 g, and 1.8 g*.

g-level	Contrast	Variable	Estimate	SE	DF	*t*	*P*	Effect Size (95% CI)
0 g	Short vs. Static	Var PE	6.2	7.3	72	0.85	>0.5	0.36 (−0.67, 1.05)
		Mean PE	−0.3	2.6	72	−0.13	>0.5	−0.04 (−1.08, 1.09)
	Long vs. Static	Var PE	26.4	6.5	72	4.06	<0.001	1.53 (0.81, 2.88)
		Mean PE	−4.2	2.6	72	−1.58	0.35	−0.54 (−1.9, 0.49)
	Long vs. Short	Var PE	20.1	5.2	72	3.91	<0.001	1.12 (0.07, 2.33)
		Mean PE	−3.8	2.6	72	−1.46	0.45	−0.34 (−1.67, 0.6)
1 g	Short vs. Static	Var PE	3.5	7.3	72	0.48	>0.5	0.15 (−0.87, 0.94)
		Mean PE	−0.7	2.6	72	−0.26	>0.5	−0.12 (−1.04, 0.84)
	Long vs. Static	Var PE	9.5	6.5	72	1.46	0.45	0.48 (−0.49, 1.68)
		Mean PE	−1.8	2.6	72	−0.70	>0.5	−0.25 (−1.03, 0.67)
	Long vs. Short	Var PE	6.0	5.2	72	1.17	>0.5	0.47 (−0.54, 2.53)
		Mean PE	−1.2	2.6	72	−0.44	>0.5	−0.13 (−0.87, 1.29)
1.8 g	Short vs. Static	Var PE	6.7	7.3	72	0.92	>0.5	0.25 (−0.88, 1.08)
		Mean PE	2.6	2.6	72	0.98	>0.5	0.31 (−0.67, 1.09)
	Long vs. Static	Var PE	19.1	6.5	72	2.94	0.013	0.73 (−0.33, 1.57)
		Mean PE	4.7	2.6	72	1.79	0.24	0.62 (−0.36, 1.2)
	Long vs. Short	Var PE	12.4	5.2	72	2.40	0.056	0.8 (0.03, 1.58)
		Mean PE	2.1	2.6	72	0.81	>0.5	0.22 (−0.76, 1.23)

*SE, standard error; DF, degrees of freedom; *t*, t-statistic; P, p-value. P values were adjusted for multiple comparisons using a Bonferroni correction. Corrections were applied to each main effect, i.e., for three contrasts. Effect size is Cohen’s *d* with bootstrap 95% confidence intervals (adjusted for three comparisons using Bonferroni correction). Note that no contrasts were performed between task conditions for reactions time because reaction times were a logical consequence of task condition, i.e., the pointing error had to be moved a shorter angular distance for static and short updating trials compared to long updating trials (for details see “Behavioral and Statistical Analysis” in “Materials and Methods” section).

**Table 2:** Contrasts examining the effects of g-level on variable pointing error (Var PE), mean direction of signed pointing error (Mean PE) and reaction time (RT) between 0 g, 1 g and 1.8 g for static (Static), short updating (Short), and long updating (Long) conditions*.

g-level	Contrast	Variable	Estimate	SE	DF		*P*	Effect Size (95% CI)
Static	0 g vs. 1 g	Var PE	−2.5	4.3	72	−0.58	>0.5	−0.18 (−1.08, 0.84)
		Mean PE	3.9	2.6	72	1.51	0.41	0.67 (−0.21, 1.44)
		RT	−0.6	0.1	162	−3.94	<0.001	−0.46 (−0.76, -0.12)
	0 g vs. 1.8 g	Var PE	−3.5	4.3	72	−0.81	>0.5	−0.25 (−1.43, 0.75)
		Mean PE	6.7	2.6	72	2.57	0.04	1.06 (0.07, 2.07)
		RT	−0.2	0.1	162	−1.28	>0.5	−0.23 (−0.55, 0.07)
	1.8 g vs. 1 g	Var PE	1.0	4.3	72	0.23	>0.5	0.12 (−0.95, 0.94)
		Mean PE	−2.8	2.6	72	−1.07	0.87	−0.47 (−1.16, 0.53)
		RT	−0.4	0.1	162	−2.67	0.025	−0.38 (−0.66, -0.02)
Short	0 g vs. 1 g	Var PE	0.3	4.3	72	0.07	>0.5	0.03 (−0.88, 0.86)
		Mean PE	4.3	2.6	72	1.64	0.31	0.54 (−0.43, 1.78)
		RT	−0.4	0.1	332	−3.82	<0.001	−0.33 (−0.58, -0.06)
	0 g vs. 1.8 g	Var PE	−3.9	4.3	72	−0.92	>0.5	−0.25 (−1.03, 0.67)
		Mean PE	3.9	2.6	72	1.47	0.44	0.45 (−0.57, 1.25)
		RT	−0.3	0.1	332	−2.71	0.021	−0.19 (−0.43, 0.05)
	1.8 g vs. 1 g	Var PE	4.2	4.3	72	0.99	>0.5	0.37 (−0.84, 1.17)
		Mean PE	0.4	2.6	72	0.17	>0.5	0.12 (−1.88, 0.92)
		RT	−0.1	0.1	332	−1.11	>0.5	−0.08 (−0.29, 0.14)
Long	0 g vs. 1 g	Var PE	14.4	4.3	72	3.37	<0.01	0.94 (0.07, 2.08)
		Mean PE	1.6	2.6	72	0.62	>0.5	0.13 (−1.04, 1.01)
		RT	−0.4	0.1	334	−3.31	<0.01	−0.25 (−0.47, -0.02)
	0 g vs. 1.8 g	Var PE	3.8	4.3	72	0.89	>0.5	0.24 (−0.79, 1.64)
		Mean PE	−2.1	2.6	72	−0.80	>0.5	−0.16 (−1.22, 0.9)
		RT	−0.3	0.1	334	−3.12	<0.01	−0.28 (−0.51, -0.04)
	1.8 g vs. 1 g	Var PE	10.6	4.3	72	2.48	0.047	0.66 (−0.21, 1.87)
		Mean PE	3.7	2.6	72	1.42	0.48	0.66 (−0.17, 1.96)
		RT	0.0	0.1	334	−0.18	>0.5	−0.05 (−0.27, 0.17)

*SE, standard error; DF, degrees of freedom; *t*, t-statistic; P, p-value. P values were adjusted for multiple comparisons using a Bonferroni correction. Corrections were applied to each main effect, i.e., for three contrasts. Effect size is Cohen’s *d* with bootstrap 95% confidence intervals (adjusted for three comparisons using Bonferroni correction).

RT was significantly affected by g-level in each task condition (static: *F*_(2,162)_ = 8.1, *P* < 0.001; short updating: *F*_(2,332)_ = 7.7, *P* < 0.001; long updating: *F*_(2,334)_ = 6.9, *P* < 0.01). Contrasts showed that subjects responded significantly faster during 0 g compared to 1 g across all task conditions (static: *t*_(162)_ = −3.9, *P* < 0.001; *d* = −0.46 (−0.76, −0.12); short updating: *t*_(332)_ = −3.8, *P* < 0.001; *d* = −0.33 (−0.58, −0.06); long updating: *t*_(334)_ = −3.3, *P* < 0.01; *d* = −0.25 (−0.47, −0.02). RTs were also significantly shorter during 1.8 g compared to 1 g for static trials [*t*_(162)_ = −2.7, *P* = 0.03; *d* = −0.38 (−0.66, −0.02)], but neither for short updating [*t*_(332)_ = −1.1, *P* > 0.5; *d* = −0.08 (−0.29, 0.14)] nor for long updating trials [*t*_(334)_ = −0.2, *P* > 0.5; *d* = −0.05 (−0.27, 0.17)]. Comparisons and effects of RT between 0 g and 1.8 g are provided in Table 2. Variable pointing error was negatively correlated with average RT (Pearson’s *r*) across all conditions at 1 g (static: *r* = −0.16; short updating: *r* = −0.18; long updating: *r* = −0.1), in static trials at 1.8 g (*r* = −0.09), and in short and long updating trials at 0 g (*r* = −0.26 and *r* = −0.33, respectively). To assess whether the effects on pointing performance in 0 g were confounded by RTs, i.e., that lower pointing performance was caused by shorter response times, we reanalyzed the effect of g-level on pointing performance by adjusting for RT. We fitted a mixed model to predict variable pointing error in 0 g that included g-level and mean RT as fixed factors and subject as a random factor (random intercept and slopes for g-level). This model confirmed the evidence of an effect of g-level after controlling for RT (*F*_(2,17)_ = 3.92, *P* = 0.04), which was qualified by a nearly significant difference between 0 g and 1 g (*t*_(17)_ = 2.62, *P* = 0.054). Note that this analysis is limited to the level of subjects and does not account for any relationship between RT and pointing error at the trial level, i.e., within subjects. This is due to the nature of the definition of variable pointing error reflecting the precision across trials in each condition. To verify the robustness of these results we also fitted a model on absolute pointing error, where we estimated the within- and between-subject effects of RT on pointing performance. As suggested by van de Pol and Wright (2009), we first determined the mean values of RT for each individual, condition and g-level to express the between-subject variation component. Next, we used within-subject centering to characterize within-subject effects by calculating the difference of each observation from the subject’s respective mean value. We then estimated the variation in both sources of variance in pointing performance using a mixed linear model with absolute pointing error as a response variable, and between- and within-individual components of RT as fixed effects. These analyses confirmed that pointing performance and RT covaried within individuals much stronger than between subjects. Trial-to-trial changes in RT predicted trial-to-trial changes in absolute pointing error within the same individual (*F*_(1,332)_ = 8.89, *P* < 0.01). In contrast, between-subject variation in reaction did not significantly predict absolute pointing error (*F*_(1,332)_ = 1.59, *P* = 0.21). The effect of g-level remained nearly significant (*F*_(2,332)_ = 2.48, *P* = 0.085) with a significant contrast for absolute pointing error between 0 g and 1 g (*t*_(332)_ = 1.97, *P* = 0.049).

## Discussion

This study investigated the acute effects of weightlessness and hypergravity using parabolic flight maneuvers on spatial updating performance in humans. Spatial updating was assessed by a virtual 3D task that required participants to encode the identity and location of objects, memorize and then update the egocentric object coordinates during a forward movement simulated by optic flow. Using different lengths of the translational movement provided a variation in updating complexity. To disentangle the effects between altered gravity conditions and changes in general cognitive performance related to the unique experimental situation, we included a static condition in the paradigm. In this condition, participants did not experience any movement, removing the need to process self-motion cues to update egocentric object positions. Since the target stimuli were presented at identical positions across all three conditions, and given that the virtual environment in the delay and retrieval was identical for all trials, all conditions are directly comparable. The delay phase, i.e., the time between encoding and retrieval of the memorized object locations, introduces noise due to working memory decay and the updating process. Given that the noise further decreases the ability to correctly memorize the target locations, we quantified the noise by calculating the standard deviation of the pointing errors for each condition and subject. Because the target locations were identical across all conditions, any pointing error would be indicative of an effect of gravity conditions.

We found that pointing error variability was increased in updating compared to static trials across all gravity conditions. The difference between task conditions was particularly prominent for long updating trials in 0 g and 1.8, where the effect reached statistical significance, suggesting an increased complexity of keeping track of object locations. Spatial updating requires to memorize egocentric object representations. This process requires to direct visual attention to the target locations, which is closely linked to working memory (Wolbers et al., 2008; Theeuwes et al., 2009; Anderson et al., 2010) and particularly demanding when objects are no longer visible and body position changes (Boon et al., 2018). Accordingly, increased errors in the long updating trials suggest the need for higher working memory and processing demands to update changing object coordinates. These findings are well in line with data reported by Wolbers et al. (2008) and Müller et al. (2018), confirming the validity of the paradigm to assess spatial updating performance.

The primary objective was to investigate the effects of weightlessness and hypergravity on spatial updating performance. We found that 0 g and 1.8 g significantly impaired performance for long spatial updating trials compared to 1g as indicated by higher variable pointing errors. This difference could not be explained by a tradeoff between pointing error and RT. Neither short updating nor static trials, which required only little or no spatial updating and therefore served as control conditions, revealed any impairments. These findings confirm our hypothesis that general cognitive performance is not impaired *per se* during weightlessness, but gravity affects distinctive cognitive domains.

Grabherr et al. (2007) compared object-based and egocentric spatial transformation tasks during parabolic flight and observed poorer performance for egocentric spatial transformation during 0 g, but no changes for object-based transformations. The authors concluded that spatial rotations of external objects can be solved by visual cues, whereas spatially updating the egocentric representation of one’s own body relies on the integration of visual information to a gravitational reference frame (Grabherr et al., 2007; Grabherr and Mast, 2010). This assumption is related to the notion that gravity provides distinct cues for sensorimotor integration and transformations of retinotopic, gravitational, and proprioceptive reference frames (Tagliabue and McIntyre, 2011, 2013, 2014). Note that the visibility of objects in the encoding phase was identical across all conditions (all objects were displayed for 1 s in each condition). The difference between static and updating conditions, and particularly long updating conditions, could be related to a change in the point of view during the encoding phase. In the static condition, the point of view and egocentric object locations remain constant. Hence, despite the disappearance of the objects, participants can still rely on the same egocentric object locations during the encoding phase. In contrast, in the updating condition the point of view changes as a result of the forward movement. This situation is aggravated in long updating trials because the object locations lie outside the visual scene most of the time due to the extended forward translation. Encoding retinotopic representations of egocentric object representations are particularly demanding when objects are no longer visible and body position changes (Boon et al., 2018). Objects that are outside the field of view (FOV) increasingly rely on input from nonretinotopic (e.g., motion-based and proprioceptive) cues. In the 0 g condition, this situation may be particularly challenging because gravity is critical for sensory transformations when visual information is lacking (Tagliabue and McIntyre, 2011, 2013, 2014).

To verify this hypothesis, we analyzed the interaction between the g-level and the visibility of the object locations. In other words, we did not assess the effect of the visibility of the objects themselves—they disappeared after 1 s in all trials—but whether their original locations remained in the FOV during the encoding phase. We ran a mixed model on variable pointing error and entered g-level and FOV as fixed factors. The factor FOV was characterized by three levels as follows. The first level comprised trials, in which both object locations remained within the FOV. The second level included trials, in which only one of the object locations remained within FOV. The third level characterized trials, in which none of the two object locations remained visible in the FOV. The interaction between g-level and the FOV on variable pointing error was nearly significant (*F*_(4,72)_ = 2.15, *P* = 0.084). A contrast analysis revealed that the interaction was driven by trials, in which none of the two objects remained constantly within the FOV. We observed a significant difference of variable pointing error between 0 g and 1 g (0 g vs. 1 g: *t*_(72)_ = 2.53, *P* = 0.041) and between 0 g and 1.8 g (*t*_(72)_ = 2.80, *P* = 0.02), but not between 1 g and 1.8 g (*t*_(72)_ = 0.26, *P* > 0.5). These data suggest that lack of gravity may have impaired the ability to update egocentric object locations when these locations are no longer in the FOV.

A methodological explanation that may have contributed to higher variable pointing error during long updating trials could be related to potential geometrical artifacts associated with the anterior-posterior distance between the target and subject’s position. Longer anterior-posterior distances would be expected to result in smaller angular errors. In static trials the average displacement was 35 m vs. 10 m for short and −10 m for long updating trials, predicting that the same positional error has a smaller effect in static compared to updating trials. Our findings, however, argue against such a confounding effect. Note that the absolute distance between short and long updating trials is identical (10 m). Accordingly, assuming that the subject responses are driven by geometrical effects the short and long updating condition should be characterized by similar errors. Second, if geometrical artifacts had confounded pointing errors, we would most certainly expect a difference between static and short updating trials. Neither of these two conditions was confirmed by our data. We did not observe any difference between static and short updating trials, and long updating trials were characterized by substantially larger errors than short updating trials. We are therefore confident that our findings are not confounded by geometrical effects associated with the anterior-posterior distance between targets and the virtual subject position.

In summary, our data show support for the notion that the absence of gravity affected the ability to encode egocentric object representations when their locations are outside the FOV. However, this may not fully account for the increased variability of pointing error during 0 g, and particularly 1.8 g. A broader alternative explanation for the vulnerability of spatial perception upon entry into microgravity and hypergravity observed in the present study and other experiments could be related to a mismatch between semicircular canals vs. otolith signals (Glasauer and Mittelstaedt, 1998). Our task was designed to require participants to integrate visual flow and egocentric object vectors in working memory, which was shown to be attributed to the precuneus (Wolbers et al., 2008). The precuneus receives input from various vestibular and multi-sensory cortical areas, such as the intraparietal sulcus, the inferior parietal lobe, and the parietal operculum (Leichnetz, 2001). A recent study showed that a single galvanic vestibular stimulation resulted in a positive blood-oxygen-level-dependent (BOLD) response in the precuneus (Della-Justina et al., 2015), suggesting a direct relationship between the vestibular system and the precuneus. It is possible that the impaired spatial updating performance during 0 g was caused by the unloading of the otoliths, lacking a critical reference cue (Glasauer and Mittelstaedt, 1998) for integrating visual information to efficiently update egocentric object locations during the presence of motion. Given the strong projections of the vestibular system to the precuneus, possibly, the reduced gravity affected the precuneus and its ability to perceive self-motion cues to update the stored egocentric object representations. This is also in line with clinical findings in patients with vestibular lesions, showing that vestibular signals are necessary for other sensory cues to be properly integrated and play a critical role in the representation of extrapersonal space (Borel et al., 2008). Support for this assumption comes from previous behavioral and neurophysiological data obtained during and after the parabolic flight. Klein et al. (2019) reported that cortical current density in the parietal area was decreased in 0 g compared to 1 g, and these reductions were not related to hemodynamic changes (Klein et al., 2019). Van Ombergen et al. (2017) performed resting-state functional magnetic resonance imaging (MRI) before and after the parabolic flight and found a lower intrinsic connectivity in the right temporoparietal junction after the flight exposure. Clément et al. (2016) recently investigated the effects of weightlessness during parabolic flight on egocentric distance perception (Clément et al., 2016). They found that egocentric distance using self-motion is overestimated during weightlessness for distances less than 4 m, and underestimated for distances over 4 m. Although these findings remain inconclusive regarding the direction or interaction of the relationship between gravity and distance perception, they suggested that altered gravity levels can change the perceived representation of distance. Data from spaceflight have also revealed that depth perception is altered during microgravity (Clément and Demel, 2012). The current experimental setup cannot verify whether poorer performance in spatial updating was mediated *via* impairments in spatial perception and orientation or other mechanisms. Long-duration studies on the ISS and future exploratory space missions could help to better understand the role of such mechanisms using specific tasks assessing spatial orientation (e.g., line orientation test) and more complex and integrative tasks of spatial cognition such as spatial updating, path integration, and spatial navigation.

Notably, our data should be interpreted in light of some confounders associated with the parabolic flight maneuver. Clément et al. (1989) showed that the gaze position can shift downwards in 0 g and shift up in 1.8 g, potentially affecting visual perception. Given that the stimuli were presented at a distance of about 50 cm at a viewing angle of approximately 15° (normal line of sight), we do not expect that the gaze position affected our data. Moreover, the target stimuli were presented at identical positions across all three conditions and the virtual environment in the delay and retrieval was identical for all trials. Hence, the selective impairment in spatial updating but not control conditions in microgravity argues against a mere conflict between head posture and gravitational acceleration, which is essential for encoding the spatial orientation of the human body in space (Cullen and Taube, 2017). Degradations in visual acuity associated with altered optokinetic responses during changing gravity conditions may also account for changes in neuro-vestibular performance. Experiments on the Mir station have shown that vertical pursuit movements are strongly affected. André-Deshays et al. (1993) showed that upward visual pursuit was largely suppressed in weightlessness, whereas less dramatic effects were reported for downward visual pursuit (André-Deshays et al., 1993). It has been suggested that this degradation of performance relates to the altered otolith input in weightless conditions (Lackner and DiZio, 2000). These results are also supported by parabolic flights, showing a tendency for upward slow phase velocity to be attenuated and downward optokinetic responses to be augmented (Clément et al., 1992a,b). Since otolith signaling drives not only vertical but also torsional eye movements, it may not be surprising that parabolic flight has also been shown to induce torsional misalignments during 0 g and 1.8 g (Markham et al., 2000; Beaton et al., 2015). To better understand the effects of changing g-levels on task performance, future studies should consider tracking eye movements during neurobehavioral testing. Other research has also shown that gravity affects visual processing. Cheron et al. (2014) recorded visual evoked EEG potentials (VEP) during a virtual spatial navigation task in astronauts on ISS. They showed that VEP potentials were preserved in weightlessness for the control condition (2D checkerboard), but not the 3D stimuli. They also reported changes in EEG spectral power for the 3D stimuli, indicative of a modulation of primary visual signals. Given the nature of the virtual 3D paradigm used in the present study, it is, therefore, possible that the effects on pointing performance observed in 0 g and 1.8 g are at least somewhat explained by a suppression of feedback or top-down mechanisms acting on the primary visual cortex. We acknowledge that scopolamine, a muscarinic acetylcholine antagonist, can dampen arousal and impair sensorimotor function, working memory, and spatial cognition (Blokland et al., 2016; Svoboda et al., 2017). However, all inflight testing was performed after the administration of scopolamine. Given that the task conditions were randomized across parabolas it is very unlikely that pharmacokinetic effects can account for the present findings. For the same reasons, it is rather unlikely that affective changes, previously suggested to be related to changes in electrocortical activity during microgravity (Schneider et al., 2008; Brümmer et al., 2011), account for the impaired spatial updating performance observed in 0 g.

It is important to acknowledge some limitations regarding our experimental design. First, to avoid potential effects associated with time-keeping mechanisms (Riemer et al., 2014), the duration of all trials was kept constant at 3 s. As a result, movement speed was correlated with the length of the traversed distance in updating trials. Given that the time between encoding and retrieval was identical across task conditions, longer moving distances were necessarily combined with a higher speed, which represents a potential confound. It is therefore also possible that the g-related variation in velocity-to-position integration could be the mechanism of the observed increases in variable pointing error. Second, the pointing indicator shown in the retrieval phase was always aligned with the direction of the virtual forward movement to reduce error variance. Consequently, RTs were correlated with movement times because the pointer had to be moved larger angular distances for updating trials. To compare conditions, future studies may consider using a joystick to log responses by pointing to the target object. With longer exposures to weightlessness, i.e., suborbital flights of experiments on the ISS, it is also feasible to use a range of pointer orientations during the start of the retrieval phase and elucidate the effect of movement speed on spatial updating performance. Finally, the task was presented on a 2D screen, creating a limited immersive virtual experience that can increase errors associated with the initial encoding phase of the objects and the perception of locomotion. However, these inaccuracies were constant across all conditions because the target objects and their locations were identical across all gravity conditions. We only included straight forward translations. This was necessary to prevent nausea in the subjects, which could be caused by passive virtual movements along curved paths. Furthermore, movements along curved paths would also introduce a potential source of ambiguity for the task, as individuals have different preferences for the frame of reference against which to make their judgments (Gramann et al., 2006). Irrespective of the degree of immersion, passive or virtual information on locomotion can be interpreted differently with respect to actual movements (Cullen and Taube, 2017). For instance, the perception of self-motion can be underestimated when no actual movements are performed (Frissen et al., 2011). The present experimental paradigm used a visual flow to simulate a forward movement, potentially lacking proprioceptive or vestibular information about locomotion. However, these cues were absent across all conditions and general differences in spatial updating performance between self-propelled, passive or no locomotion may not necessarily question our findings. Nevertheless, the present data should be interpreted cautiously concerning natural movements that provide both visual and body-based self-motion cues.

Taken together, our data show that performance for long spatial updating trials is impaired during weightlessness and hypergravity. We also demonstrated that general cognitive performance is not affected *per se* as indicated by the lack of any effects in the static control task condition, suggesting that gravity levels affect those areas of the brain that have strong projections to the vestibular system. We suggest that the discrepancy between canal and otolith signaling associated with altered gravity conditions may have played a critical role in the impaired pointing performance observed in the present study because of the various afferents between the precuneus and other parietal brain areas associated with spatial abilities and the vestibular system. The adverse effects of g-levels on performance for long spatial updating trials observed in the present study could be relevant for spaceflight because spatial updating is a critical skill for navigation, particularly when visibility is poor or objects go out of sight such as during extravehicular activities. Moreover, the effect could be exacerbated because our data were collected in a seated position. Future studies should compare the effects of seated vs. free-floating conditions on pointing error and determine at which g-threshold decrements in spatial updating performance emerge.

## Data Availability Statement

The data that support the findings of this study are available from the corresponding author (AS), upon reasonable request.

## Ethics Statement

The studies involving human participants were reviewed and approved by the Ethics Committee of Charité—Universitätsmedizin Berlin, the European Space Agency (ESA) medical board, and the French Ethics Committee—Comité de Protection des Personnes (CPP Nord-Ouest III) and authorized by the French Competent Authority (ANSM). The participants provided their written informed consent to participate in this study.

## Author Contributions

AS designed and directed the project, analyzed all data and wrote the manuscript. AW and KB helped supervise the project and performed data collections. PD and SB provided technical and scientific expertise in designing parabolic flight experiments. PD also allowed performing a feasibility study as part of the 62nd CNES parabolic flight campaign that provided the basis for the design and implementation of the present experiment. PD also obtained Ethical approval from the French Ethics Committee—Comité de Protection des Personnes (CPP Nord-Ouest III). SB served as the flight surgeon. He performed a final medical check and administered the scopolamine. TW designed the original paradigm, MR and AS adapted it for spaceflight. H-CG and SK provided critical feedback and contributed to the interpretation of the results. All authors discussed the results and contributed to the final manuscript.

## Conflict of Interest

The authors declare that the research was conducted in the absence of any commercial or financial relationships that could be construed as a potential conflict of interest. The handling Editor declared a past co-authorship with one of the authors SB.

## Footnotes

^1^www.novespace.fr
